# Over a millon Creatine Kinase due to a heavy work-out: A case report

**DOI:** 10.1186/1757-1626-1-173

**Published:** 2008-09-20

**Authors:** Pablo Casares, Jorge Marull

**Affiliations:** 1 10 Amsterdam Ave. Apt 306, zip 10023, New York, NY, USA; 2 515 59th St, Apt 25C, 25th FL, zip 10019, New York, NY, USA; 3 Department of Medicine, St Luke's Roosevelt Hospital Center, Columbia University College of Physicians and Surgeons, New York, NY, USA

## Abstract

Rhabdomyolysis induced by exercise is a very well known entity, several cases has been reported in the literature related with strenuous activities, weight lifting, marathon running, overexertion in an untrained person, knee bends, etc. We reported an interesting case of exercise-induced rhabdomyolysis in a 25 year old Hispanic male, after resuming his regular physical activity, with the highest creatine kinase described in the literature, successfully treated with aggressive hydration only and no complications.

## Background

Presentation of rhabdomyolysis is characterized by a triad of symptoms that include myalgias, pigmenturia and muscle weakness; accompanied with an elevation of serum muscle enzymes. [1,2]. A broad spectrum of complications and clinical manifestations have been described, from asymptomatic cases up to electrolytes imbalances, acute renal failure, compartimental syndrome and life threatening conditions followed by death.[1,3,4]. However, this presentation is not always evident and special importance should be given to the clinical history, including medications, toxic substances, illicit drugs, alcohol consumption, inflammatory and infectious process, metabolic and genetic disorders among others. Because an early recognition of this entity and a prompt intervention may prevent a serious injury or even death.[1,5]

## Case report

This is a case of a 25-year-old Hispanic male presented to the Emergency Service with one-day history of dark urine and left thigh swelling. Two days prior to his complains, after having no training for the last six months, he decided to resume his physical activity. Without previous warming-up he began with a very heavy leg work-up. He also forced his left leg more than his right leg, the reason for that was 12 months earlier he had underwent surgery of his left knee due to an anterior cruciate ligament injury.

After 48 hours he noticed his urine became dark and his left thigh got swollen. He denied any past medical history apart from the surgery, he was taking no medications and he also denied any alcohol, drugs, steroids, hormones, dietary supplements consumption or injections on his lower extremities. Social, family and sexual histories were unremarkable. The physical exam revealed a mildly left thigh swelling, compared to his right thigh, and tenderness over his left quadriceps to deep palpation. Neurological exam was normal, and pulses were present in both extremities; also a compartimental syndrome was excluded with a Doppler Ultrasound. Chemistry analysis (Table 1), Urinalysis (Table 2) and Urine toxicology screening (Utox) was negative for alcohol, amphetamines, barbiturates, benzodiazepines, cocaine, methadone, opiates and phencyclidine.

**Table 1:** 

**Chemistry**				**Hematology**	
Sodium	142	Albumin	3.4	WBC	9.5
Potassium	4.0	A/G Ratio	1.3	Hemoglobin	14.8
Chloride	110	Bilirubin, Tot	0.9	Hematocrit	42.9
C02	31	Bilirubin, Dir	0.0	Platelet	270
Urea Nitrogen	9.0	Alk. Phosph.	54	PT	15.0
Creatinine	1.0	AST	2292	INR	1.2
Glucose	69	ALT	555	PTT	28.2
Magnesium	1.8	C K	1454952		
Calcium	7.8	CK-MB	12.8		
Phosphorus, In	3.7	Ck-MB index	0.0		
Uric Acid	4.2				
Protein Total	6.0				

Ref: Creatine Kinase (CK), Aspartate aminotransferase (AST), Alanine aminotransferase (ALT), white blood cell count (WBC).

**Table 2:** 

**Urinalysis**			
**Color**	Light Yellow	WBC, Urine	0–5/HPF
Appearance	Clear	RBC, Urine	0–3/HPF
Specific Gravity	1.015	Squamous Epithelial cells	Present A
pH – UR	7.0		
Protein, Urine	Negative		
Glucose, Urine	Negative		
Ketones	Negative		
Bilirubin	Negative		
Blood, Urine	Large A		
Nitrites	Negative		
Urobilinogen	0.2		
Leukocytes esterase	Negative		

Due to the clinical scenario and the value of creatine kinase (CK) obtained 1,454,952 UI, the patient was diagnosed of exertion-induced rhabdomyolysis. Also an increased in his aminotranferases enzymes was observed Alanine Aminotranseferase (ALT) 555, and Apartate Aminotransferase (ALT) 2292. Laboratory results were repeated 4 hours apart due to the electrolyte imbalances commonly seen in this pathology, showing a CK of 1,423,878 and normal creatinine and potassium. He was treated with a vigorous hydration, intravenous fluids (IV) of 0.9% NaCl, at a rate of 500 ml/hr, reaching a urinary output > 200 ml/hr. During his clinical course he presented no complication, 12 hours later the CK concentration was 321.244 UI, on day 2 CK was 299.148, on day 3 the CK decreased up to 181.196. After four days of IV fluids, his CK was 95.870, creatinine 0.9 and his thigh swelling was solved. The patient was discharged on day 5 with a CK of 52.476, creatinine of 0.9 and monitored as outpatient every 2 days, checking his CK and renal function. After 8 days his CK was less than 1000 with a normal creatinine 0.9.

## Discussion

Serum level of CK is considered the most reliable indicator of rhabdomyolysis. CK levels with a range from 10.000 to 300.000 are common in exercise induced rhabdomyolysis, in this case e the CK was more than 1.400.000, being this case the highest CK reported in the medical literature due to physical activity in an otherwise healthy person.[6]

Rhabdomyolysis may occur with normal physical activity, but outrageous exercise can provoke a massive rhabdomyolysis. The following factors have been associated with this condition, impaired sweating, untrained persons, exertion in extremely humid conditions, persons with sickle cell trait who exercises at high altitude, enzymatic deficiencies.[7-9]

Different incidences of acute renal insufficiency ranging from 17% to 40% have been reported, but the real incidence of rhabdomyolysis due to vigorous physical activity seems to be unknown [10,11]. Senert et al, in a retrospective cohort study where 35 patients were included evidenced no acute renal failure in all of them, concluding that exercise-induced rhabdomyolysis in persons without previous renal impairment might have a lower incidence of acute renal failure than other forms of rhabdomyolysis.

The treatment of this medical condition consists in preserving the renal function, managing the electrolytes imbalances in order to prevent cardiac arrhythmias, disseminated intravascular coagulation, etc. Intravenous hydration (IV) is the corner stone of its treatment, Sauret et al. recommends IV hydration with normal saline trying to reach an urinary output of 300 ml per hour until the myoglobinuria has ceased, and should be kept such a high rate of IV hydration until CK level decreases to or below 1.000 UI.[5] Some authors, like Zager RA, recommends the use of sodium bicarbonate to alkalinize the urine and prevents the deposition of myoglobin casts, decreasing the toxicity into the tubules.[12] The use of manitol is controversial and is mostly supported by experimental data in animals and some retrospective studies.[13] Those patients who don't respond and develop acute renal failure, with inadequate control of hyperkalemia, volume overload or uremia, hemodialysis should be started.

## Conclusion

In this case report, the patient was treated with intravenous fluids only, without any complications. Apart from that, IV fluids were stopped when the CK level was more than 50.000 IU and managed as an out patient with frequent monitoring. Sauret et al recommends that IV fluids should be continued until CK is less than 1000 IU and myoglobin in urine is negative, because the nephrotoxicity induce by aciduria and myoglobinemia has been experimentally demonstrated, when the pH is less than 5,6 myoglobin suffers dissociation into globulin and hematin. Heme iron may induce the formation of toxic free radicals, and when urine pH decreases below 5.0 myoglobin solubility decreases as well, allowing myoglobin cast formation with a subsequent tubular deposition. This situation has been correlated with the development of acute renal failure.[14]

It is very well known that most of physicians try to keep these patients in the hospital and discharge them when the CK is between 5.000 and 10.000. However, there is no trials to support this position, also there is no consensus regarding the safest CK level to determine when to stop the IV hydration or when is safe to discharge a patient if high serum CK levels are found. Treatment is another controversial point as we mentioned before, showing that all measures should be directed to prevent and treat early and late complications. Obviously this is one case only, and no definitive conclusions can be done, but it might suggest that in those patients without comorbidities presenting with exertional-rhabdomyolysis the CK is not a predictor factor of further complications such as renal insufficiency; allowing physicians a safe management of this patients in an out patient setting, and also avoiding an unnecessary hospital stay. To confirm and support this supposition and determine when is safe to discharge them more studies need to be conducted.

## Competing interests

The authors declare that they have no competing interests.

## Authors' contributions

PC was a major contributor in writing the manuscript, analyzing the data, and collecting the information in the outpatient setting. JM wrote the abstract and made a contribution in the conclusion. Both authors read and approved the final manuscript

## Consent

The following case was evaluated and approved by the Institutional Review Board (IRB), inform consent is not required because the patient identity is not identified in the article. However, written informed consent was obtained from the patient for publication of this case report and accompanying images. A copy of the written consent is available for review by the Editor-in-Chief of this journal.

Atenucci Research Pavillion 432 West 58^th ^Street – Room 207, phone 212-523-4370, Fax: 212-523-7442. 
